# Supplementary material for: Are carbon emissions trading and green financial instruments synergistic? -Comprehensive quantitative research based on content analysis
Source: PLoS One. 2024 Mar 7;19(3):e0298601. doi: 10.1371/journal.pone.0298601 (PMC10919601; doi:10.1371/journal.pone.0298601)
Supplement: S1 Data — (ZIP) [file pone.0298601.s001.zip › Data/地级市Z_F工作报告.zip!/╔╜╢1⁄2/╝├─■╩╨/└┤╫╘╣1⁄2╓┌║┼í╛┬φ┐╦╩2╛▌═°í┐_2016.docx]

各位代表：

现在，我代表济宁市人民政府向大会报告工作，请予审议，并请市政协委员和其他列席会议的同志提出意见。

一、“十二五”时期工作回顾

过去的五年极不平凡，在中共济宁市委的坚强领导下，全市上下团结拼搏，扎实苦干，圆满完成“十二五”规划目标任务。全市地区生产总值达到4013.1亿元，年均增长10.2%；一般公共预算收入达到368.6亿元，实现翻番；固定资产投资达到2891亿元，年均增长19%；社会消费品零售总额实现1911亿元，年均增长13.8%；城乡居民收入年均分别增长10.6%、13.1%。县域GDP完成3995.6亿元，五年增长65.7%；所有县市区一般公共预算收入均实现倍增。

（一）转型升级步伐加快。三次产业比例由2010年的12.6:53.3:34.1调整为11.3:47.3:41.4。实施“百千亿”产业培植工程，完成技改投资3222亿元，累计新增规模以上工业企业1220家、达到2631家。转型升级“十大产业”销售收入占比达到65%，非煤产业、制造业增加值占比分别达到79.3%、69.8%，高新技术产业产值累计完成6166亿元、占比提高9个百分点。9家企业销售收入过百亿，4家企业进入中国企业500强。信息产业实现主营业务收入480.1亿元、同比增长50%，惠普、中兴、甲骨文等一批IT产业大项目集群式落户，全市在建重大信息产业项目61个，总投资416亿元。服务业发展提质增效，骨干企业主辅业务分离基本完成，2015年新增规模以上服务业企业296家，居全省首位，达到1407家。五年实现服务业增加值6613.3亿元、年均增长10.8%，2015年完成税收216亿元、占比达到47.7%。粮食生产实现“十二连增”，新增新型经营主体1.5万户。全国小农水重点县实现全覆盖。粮食高产创建经验在全国推广。济宁农高区、邹城农科园晋升为国家级农业科技园区。

（二）改革开放持续深化。市县政府机构改革顺利完成，事业单位配套改革分步实施。探索建立“四项清单”制度，清理规范中介组织，取消压减行政审批458项。商事制度改革成效明显，2015年新登记市场主体13.8万户，累计达到46.3万户，是五年前的2倍。财税金融和投融资体制改革扎实推进，市县两级政府预决算、部门预决算和“三公”经费预决算全部公开。设立10亿元产业引导基金，投融资规模达到406亿元。强化金融对实体经济支持，五年投放信贷资金1357.4亿元。企业规范化改制全面实施，35家中小企业在资本市场挂牌。农村土地承包经营权确权登记颁证、集体林权制度改革基本完成。医药卫生体制改革不断深化。对外开放成效显著，2015年进出口总额增长3.9%，高于全省16.6个百分点，五年直接利用外资41.4亿美元、增长108%，对外投资44.6亿美元、居全省前列，48家世界500强企业落户我市。创新能力显著增强，8家国家、省科研院所在济宁设立分支机构，建成国家级重点实验室3家、省级以上工程技术研究中心71家。济宁高新区获批“中国产学研合作创新示范基地”，如意集团纺纱工程技术研究中心成为我市首家国家级研发平台。

（三）对上争取实现重大突破。资源型城市可持续发展试点得到国家发改委同意，采煤塌陷地生态修复治理列入国家试点项目，我市获批建设100万千瓦光伏发电示范基地，耕地保有量核减取得阶段性成效。曲阜文化建设示范区进入国家、省“十三五”规划，教育部、国家汉办在曲阜设立孔子学院总部体验基地，中组部在济宁设立干部政德教育基地。我市被确定为国家生态保护与建设示范区、山东省信息技术产业基地、宽带中国示范市、国家级信息消费试点市。

（四）城乡面貌显著变化。常住人口城镇化率达到52.75%，比五年前增加9.8个百分点。实施行政区划调整，市区面积达到1534平方公里，人口增加到177.5万，进入国家Ⅱ类大城市行列。中心城区累计实施473个重点项目、完成投资480亿元，改造棚户区1547万平方米、老旧小区188.3万平方米，建成经济适用房2.4万套、公租房1.5万套，新增供热面积1546万平方米、燃气管道用户7.1万家。都市区融合发展步伐加快，泗河全域开发、“三河六岸”改造稳步推进，一批主干路网实现贯通，城际公交线路达到13条，开通了市区至曲阜高铁站快速公交。“五城同创”取得积极进展，城市管理水平不断提高，全国绿化模范城市创建成功，全国双拥模范城市“七连冠”通过省级考评，全国文明城市创建进入提名城市行列，国家卫生城市创建争取到申报资格，全国环保城市创建进展顺利。实施美丽乡村“三年行动计划”，硬化农村道路2.1万公里。农村环境综合整治成效明显，城乡环卫一体化实现全覆盖。建成22个美丽乡村示范片区、408个示范村，93%的村达到环境整洁村标准，2015年我市荣获“中国人居环境范例奖”。

（五）生态建设扎实推进。重拳整治大气污染，空气质量明显改善，2015年“蓝天白云、繁星闪烁”增加天数居全省第1位。完善“治用保”流域治污体系，国控省控主要河流断面水质稳定达标，两次代表山东省接受国家淮河流域治污核查均取得第一名。境内南水北调东线工程全面竣工，保证了全线安全通水。完成湿地修复60万亩，治理采煤塌陷地10.1万亩，新增造林111.7万亩，森林覆盖率达到30.2%。严格执行污染物排放许可和总量控制制度，万元GDP能耗下降17%以上，减排污染物9.9万吨，超额完成“十二五”节能减排任务。

（六）社会事业全面进步。五年民生支出1599.4亿元、占财政支出的77.1%。为民所办实事如期完成。新增城镇就业38万人，农村劳动力转移就业34万人，城镇登记失业率控制在4%以内。我市荣获全国首批创业先进城市称号。扎实做好扶贫工作，全市44万贫困人口实现脱贫。五项社会保险制度实现全覆盖，“四位一体”养老服务经验在全国推广。妇幼健康提升工程稳步推进，“先看病、后付费”诊疗模式全面实施，“万名保健医生进农户”活动常态化，出生人口性别比日趋合理。累计投入资金16.4亿元，改造校舍面积136万平方米，农村中小学营养餐补助实现全覆盖。传统文化“六进普及”、道德提升“七大工程”深入实施，市文化中心开工建设，76%的村建设了文化广场。新闻出版、广播电视繁荣发展，一批文艺精品在全国、全省获奖。高水平承办第23届省运会、第9届残运会和国际孔子文化节、世界文明论坛、世界儒学大会等重大活动。平安济宁建设成效显著，社会应急管理体系不断完善。安全生产形势持续稳定，连续多年受到省政府表彰。社会治理创新、信访“双基”工作扎实推进，“社区六进”实现全覆盖，我市被评为全国社会管理综合治理优秀市。

（七）民主法制建设不断加强。主动接受人大依法监督、政协民主监督，办理人大代表议案建议646件、政协提案2323件，代表、委员满意率分别达到98%、98.2%。完善重大行政决策程序，优化政务服务流程，政府工作效率明显提升。普法和依法行政扎实推进。深入开展党的群众路线教育实践活动、“三严三实”专题教育，严格落实中央“八项规定”，持续强化行政监察和审计监督，驰而不息纠正“四风”，政风行风明显改善。圆满完成第三次经济普查。对口援疆工作取得新成效。人民防空、民族宗教、外事侨务、住房公积金、粮食、物价、地震、气象、水文、档案、史志、文物、红十字和残疾人事业等各项工作取得新成绩。

各位代表，五年来，我市积累了坚实的发展基础，创造了一系列发展机遇，特别是2013年习近平总书记视察济宁并发表重要讲话，为我市加快发展指明了方向。“十二五”时期发展的每项成绩，都是党中央、国务院和省委、省政府及市委正确领导的结果，是市人大、市政协和社会各界大力支持的结果，是全市上下努力拼搏的结果。在此，我代表市政府，向全市各族人民、各界人士、驻济解放军指战员、武警官兵和公安干警致以崇高敬意！向关心支持济宁发展的港澳台同胞、海外侨胞和国际友人表示衷心感谢！

各位代表，五年来的实践使我们深刻体会到，推进经济社会又好又快发展，必须坚决贯彻落实中央和省委、省政府及市委的决策部署，自觉接受人大、政协监督，积极争取方方面面的支持。必须转变发展理念，适应引领经济发展新常态，大力推进结构转型、产业升级，坚定不移地走转型发展之路。必须抓住用好机遇，从济宁实际出发，做好与上级政策对接融合的文章，真正使机遇优势转变为发展优势。必须全面深化改革扩大开放，激发大众创业、万众创新的热情，不断为发展注入新的动力。必须坚持问题导向，找准薄弱环节和问题短板，分类施策，精准发力，进一步增强发展的协调性。必须增进人民福祉，优先增加民生投入，提高公共服务能力，使发展成果更多更公平的惠及全市人民。必须扑下身子真抓实干，认真践行“三严三实”，不畏难、不懈怠，不做表面文章，以踏石留印、抓铁有痕的作风抓落实促发展，奋力开创济宁改革发展新局面！

我们也清醒地认识到，我市经济下行压力依然较大，部分行业和企业经营困难，发展的质量效益还不够高；产业结构不合理，重化工业占比偏高，新兴产业还没有形成规模优势，转方式调结构任务艰巨；创新能力不强，高层次人才缺乏；区域发展不平衡、不协调，城镇化水平相对滞后；节能减排、环境保护压力较大；政府机关及个别工作人员“不严不实”和“庸懒散”问题依然存在。对这些问题，我们将认真对待，努力加以解决。

二、“十三五”时期目标任务和重点工作

“十三五”时期是全面建成小康社会的决胜阶段，是实现我市“四大目标”的关键时期，也是我市发展历史上重大机遇最为集中的释放期。主要预期目标是：经济保持中高速增长，地区生产总值年均增长8%左右，主要经济指标高于全省平均水平，提前实现地区生产总值和城乡居民人均收入比2010年翻一番。产业迈向中高端，战略性新兴产业产值占比达到35%左右，信息产业销售收入突破千亿元，服务业占比达到46%。新型城镇化步伐加快，常住人口城镇化率达到65%，中心城区辐射带动能力显著增强，都市区发展深度融合，乡镇公交通达率达到100%。环境治理扎实推进，节能减排约束性指标全面完成，空气质量明显改善。群众获得感持续增强，居民人均可支配收入年均增长8%以上，城镇登记失业率控制在4%以内，文化教育、医疗卫生、社会保障等公共服务体系更加健全。

实现“十三五”规划目标，必须以“五大发展”理念为引领，协调推进“四个全面”战略布局，进一步提升发展标杆、工作标准、精神境界，更加注重转方式、补短板、惠民生，努力实现有质量有效益的发展。

要坚持创新发展，把创新摆在发展全局的核心位置，全面推进理论创新、制度创新、科技创新、文化创新。以建设国家创新型城市为抓手，大力实施创新驱动战略，增强企业创新主体地位，全方位推进产品创新、品牌创新、产业组织创新和商业模式创新，通过创新促进我市资源型城市加速转型。

要坚持协调发展，完善规划对接、政策协同、产业协作机制，加快生产要素合理流动、优化配置，构建“一体两翼、三带协同、板块支撑”的发展格局。加快高速公路等重大基础设施建设，努力把全市人民县县通高速的美好梦想变成现实。

要坚持绿色发展，规划建设主体功能区，划定和严守生态红线，推行产业准入负面清单制度，提升功能区内涵式发展水平。创新资源型城市绿色发展模式，实施最严格的环境和耕地保护制度，扎实推进大气、水、土壤污染防治行动计划。构建低碳循环发展模式，建设节水型社会和海绵城市，努力做到既要金山银山，更要绿水青山。

要坚持开放发展，实施新一轮高水平对外开放，积极融入“一带一路”和中韩自贸区建设，形成开放型经济新优势。加快重点领域关键环节改革，更大力度推进简政放权，努力打造全国领先的营商环境。

要坚持共享发展，坚持发展为了人民、发展依靠人民、发展成果由人民共享，完善民生保障“十大体系”，实施系列民生工程，推进公共服务均等化，努力让人民群众生活得更加温暖、更加舒心、更加幸福！

三、2016年政府工作总体安排

今年是实施“十三五”规划的开局之年，做好今年工作，我们要按照中央和省、市委的部署要求，深入贯彻党的十八大、十八届三中、四中、五中全会精神，以习近平总书记系列重要讲话、视察山东视察济宁重要讲话精神为指引，协调推进“五位一体”总体布局和“四个全面”战略布局，牢固树立“五大发展理念”，以结构性改革引领经济发展新常态，抓住用好系列重大机遇，更大力度突破新型工业化、新型城镇化、县域经济、文化发展、生态建设“五大领域”，全力攻坚信息产业、文化旅游和城市建设“三项重点”，持续提升民生保障“十大体系”，确保实现“十三五”经济社会发展良好开局，为实现综合实力进入全省第一方阵、鲁西科学发展高地建设继续走在西部前列、确立淮海经济区中心城市地位、与全省同步全面建成小康社会“四大目标”奠定坚实基础。主要预期目标是：地区生产总值增长8%左右；一般公共预算收入增长9%左右；规模以上工业增加值增长9%左右；固定资产投资增长12.5%左右；社会消费品零售总额增长11%左右；外贸进出口、利用外资保持适度增长；居民人均可支配收入增长8.5%左右；城镇登记失业率控制在4%以内；节能减排完成省定目标。

（一）突出抓好产业转型升级。转型是实现我市又好又快发展的关键所在，必须实施精准的产业政策，加大结构性改革力度，推动产业向中高端迈进。

全面提升现代农业发展水平。一是构建新型农业经营体系。稳步推进土地经营权规范有序流转，引导多种形式适度规模经营，大力培育各类农业经营主体。年内培育新型职业农民3000名以上，新增市级重点农业龙头企业50家。创新社会化服务机制，推广大田托管模式，新增面积50万亩以上。依托“云农场”等电子商务平台，实行线上线下农资交易、农产品经营，拉长农业产业链和价值链。二是构建农业支持保护体系。建立农业投入稳定增长机制，完善农业补贴制度，优化补贴支持方向。加大种业创新力度，完善5个商业化育种中心，建成45万亩种子标准化基地。三是构建农产品安全监管体系。加快国家现代农业示范区创建步伐，规范建设20处市级示范园区。积极创建农产品质量安全示范县，新认证“三品一标”60个。四是构建农业可持续发展体系。落实藏粮于地、藏粮于技战略，实施高标准农田规划建设，新建20万亩高产创建示范方，粮食产量稳定在100亿斤以上，切实保障粮食安全。大力调整农业结构，发展高价值经济作物和高效生态农业，促进粮经饲统筹、农林牧渔结合、种养加一体发展。扎实推进国家农业科技园区建设。五是构建安全生态大水利体系。完成泗河综合开发邹城、高新区、兖州区堤防工程建设，全面启动沿线县市区防洪工程。抓好中小河流治理、引水补源入济等项目建设，完成农田水利工程5700项，建成“旱能浇、涝能排”高标准农田23万亩，治理水土流失面积60平方公里。

加快推进工业经济提质增效。一是打造撬板，培植壮大信息产业。实施信息产业跨越发展“三年行动计划”，以建设省信息技术产业基地为牵动，提速推进宽带中国示范市、信息消费试点市、全光网示范市、智慧城市试点“四城同建”，加快惠普、中兴等专业园区建设，深化与中科曙光、IBM的战略合作，建设智慧城市云平台和鲁西南数据中心。深度对接华为、阿里巴巴等国际知名IT企业，争取更多行业巨头入驻济宁。集中扶持100家重点信息产业企业，使其成为全市经济增长的新引擎。二是厚植长板，改造提升传统产业。以供给侧结构性改革为引领，实施工业转型升级行动，落实优势传统产业提升计划，加快骨干企业技术改造、设备更新，年内完成技改投资900亿元。实施质量强市战略，推进“好品济宁”建设。加速“两化”融合，重点培育10家示范企业，鼓励企业运用物联网、云计算、大数据等现代信息技术，提升传统产业自动化、智能化水平。分类制定扶持煤炭、化工和工程机械等行业转型发展政策措施，对丧失自我修复能力的企业，通过兼并重组、债务重组、破产清算等方式，倒逼企业转型、产业升级。三是补齐短板，加速发展高端制造业。紧密对接“中国制造2025”，按照十大产业升级方向和任务目标，引进发展智能整机装备、系统总成、生物医药等高端产业，引导企业与国内外科研院所加强技术协作，增强竞争实力。继续实施中小企业“百千万培训”工程，采取政府购买服务方式，提升中小企业公共服务水平，培育一批“专精特新”制造业产业集群。

着力提升服务业规模档次。一是强化发展新支撑。分行业制定服务业发展导则，降低准入门槛，优先保障用地。抓好总投资660亿元的30个重点园区、重点项目建设，加快打造现代服务业聚集示范区。二是培植发展新业态。顺应互联网经济发展趋势，实施电商培育、电商进村计划，建设一批特色电商平台、电商镇村服务站，电子商务交易额突破1000亿元。大力发展信息服务、教育培训、医疗健康、社区家政、社会养老等服务业。以文化旅游资产整合为抓手，推进旅游资源市场化运作，深化与知名旅游集团的战略合作，加快景区整体开发运营，高标准建设一批重点旅游项目。鼓励发展乡村旅游、研学旅游。三是拓宽发展新内涵。推动生产性服务业与工业加速融合，着力发展研发设计、软件开发、检验检测等产业，促进生产性服务业向专业化和价值链高端延伸。发挥运河航道优势，大力发展铁水联运物流，规范发展快递物流业，建设商贸物流园区，打造重要物流节点城市。积极引进各类金融机构，加快发展股权投资、产权交易、产业基金等新兴金融业态。年内服务业增加值、税收占比均提高1个百分点以上。

（二）多措并举培育发展新动能。突破体制机制障碍，增强发展动力活力，培育新的经济增长点。

加快招商引资和项目建设。积极做好国家、省重大工程包和政策性贷款争取工作，力争更多项目纳入上级目录。开展项目建设、招商引资突破年活动，创新招商体制机制，突出重点区域、重点产业、重点项目，科学编制招商产业地图、企业地图和项目方案，强化精准对接和要素支撑，务求取得更大成效。年内每个县市区至少落地2个以上过10亿元产业大项目，新上一批亿元以上好项目。

着力提高科技创新能力。主动衔接国家“互联网+”行动计划和新兴产业“双创”三年行动计划，实施重大技术创新工程、企业创新自强工程，开展企业创新转型试点，鼓励企业建立研发机构，加强产学研合作，构建产业技术创新联盟，推动跨领域跨行业协同创新，年内新增工程技术研究中心50家、企业技术中心38家，新认定高新技术企业30家，全社会研发投入增长12%以上。推进惠普软件产业国际创新园加快发展。以科技成果使用权、处置权和收益权“三权”下放为重点，加快科技成果转化。实施人才强市战略，完善高层次人才招聘、考核选拔、社会保障制度，以企业为中心，引进一批泰山学者、国家“千人计划”高层次人才，全力打造“人才智力高度聚集、科技金融高效融合、创新平台优势凸显、创业环境国际接轨”的人才管理改革试验区。

全面深化各项改革。以“四张清单、一个平台”为主要架构，加快建立完善政府权力运行体系，完成省市县网上政务服务平台三级互联。进一步简政放权，再取消和下放一批行政审批事项，积极推进并联审批，完成中介机构清理规范工作。深化财税金融体制改革，牢固树立过紧日子思想，优化支出结构，提高财政资金使用效益。健全税收保障机制，推进联合办税，加快建设综合涉税信息管理平台，利用互联网、大数据等手段，抓好主体税种、重点税源的征管。继续清理财政专项资金，扩大涉农专项资金整合试点范围，规范财政转移支付。全面完成营改增任务。积极推广PPP模式，拓宽政府与社会资本合作领域。发展壮大地方金融机构和新兴金融业态，推进新型农村合作金融试点，加快多层次资本市场建设，建立融资性担保机构风险补偿机制，提升金融普惠程度和服务实体经济能力。加强对担保圈、民间借贷等信用风险监测，严厉打击非法集资和逃废金融债务行为，防止发生区域性系统性金融风险。深化国有企业改革，以市管煤炭企业为重点，完善国有资产管理体制，建立以管资本为主的国有资产监管模式，规范企业经营决策行为，实现资产保值增值。加快企业规范化改制，提高直接融资能力，年内争取10家以上企业挂牌上市。抓好农村土地确权登记颁证成果验收和应用，推进农村产权交易服务平台建设，加快农村集体产权制度改革步伐。深化供销社综合改革。

努力提高对外开放水平。按照“优进优出”的思路，推动我市产品、技术、服务全产业链出口，加大先进技术、关键设备和重要零部件进口。提升机电、造纸和农产品等重点出口产品附加值，支持高技术企业产品向高端配套发展，打造国际知名品牌。实施“千企百展”行动，引导企业参加境内外知名展会，大力发展跨境电子商务，帮助中小企业借助电商平台抓订单拓市场。加快济宁综合保税区申报工作。主动参与“一带一路”战略，引导工程机械、钢铁、水泥等优势传统行业“走出去”，带动大型成套装备和劳务输出。

增强县域经济发展活力。实施县域经济提升“三年行动计划”，强化对县域经济尤其是经济薄弱县的帮扶，在项目建设、基础设施建设、转移支付、金融支持等方面加大扶持力度。加快推进各类开发区、经济园区转型升级，积极培育各具特色的新型专业园区，完善公共设施配套，增强园区综合实力，形成强县领跑、弱县进位、竞相发展、全面崛起的县域发展格局。

（三）加快新型城镇化建设。继续实施城镇化追赶战略，做大做优中心城区，促进都市区深度融合，推进小城镇和美丽乡村建设，年内常住人口城镇化率提高2.5个百分点。

加快建设精致城市。一是实施规划引领工程，树立“精明增长”、“紧凑城市”理念，完善“3+7”城市规划体系，年内实现中心城区控规编制全覆盖。强化规划管控，以规划引领中心城区一体发展，加快产业项目和基础设施建设，加速任城区、高新区与兖州区对接融合，积极培育太白湖新区、经济技术开发区高科技产业和新兴产业，加快教育、医疗等公共基础设施配套。二是实施棚改提升工程，加快老旧小区和危房改造，将棚改纳入政府购买服务指导目录，用好货币化安置政策，提高公积金使用效率，消化商品房库存，稳定房地产市场，完成棚改任务5.2万户，改造小区严格按新标准配套完善公共服务设施。全面落实户籍管理制度改革方案，促进农业转移人口进城落户，并与城镇居民享有同等权利和义务。三是实施交通疏解工程，加快济邹路、车站西路等主干道升级改造，老城区完成火炬路北延、任兴路东延工程，启动红星路西延工程，兖州区完成荆州路北延等工程建设。推进公交优先发展战略，加快城市停车场建设，改造整治背街小巷，优化支路微循环系统，增强道路疏堵保畅能力。四是实施水系连通工程，系统开展都市区范围内塌陷地、河道水系综合整治，完成南跃进沟生态治理，实现引水入城，打造水系贯通、城水相依、碧水灵动的水城风貌。积极申报第二批海绵城市试点市。五是实施生态绿化工程，结合棚户区改造，建设不同层次的公园绿地，适度增加常绿树种。加大居民小区、单位院落拆墙透绿力度，建设一批特色鲜明、具有文化品位的小型绿地、袖珍广场，更好地为市民提供公共活动空间。启动建设济北国家森林公园，升级改造南郊动植物园、西郊苗圃等城市公园，大幅增加城市人均公园绿地面积。提升城市道路绿化水平，推进京杭大运河、泗河生态带和环城林带建设。六是实施精细管理工程，深入推进“五城同创”，全力治理“脏乱堵”。加快推进城市管理大部门制改革，构建管理、执法、服务“三位一体”城管格局。推动城市管理重心下移，发挥好属地管理作用。加快城区农贸市场标准化建设，新建改建农贸市场8个。启动建设建筑垃圾处理场，提升医疗垃圾处理能力，扩大城市垃圾场容量。加快市政养护等公共服务市场化运作。开展“工程质量管理年”活动，全面提升工程建设质量。建成数字城管指挥系统，完成主城区基础信息普查，推进城市建设管理精细化、标准化、高效化。

促进都市区融合发展。出台都市区融合发展政策法规，推动“四个一体化”实质性进展。全面展开重大基础设施建设，完成北外环东延，开工建设西二环、济宁大道西延、环湖大道东线工程，提速推进济徐高速、跨微山湖高速、跨黄河高速和济宁新机场等一批牵动性工程。加快鲁南快速铁路建设，做好城区轨道交通和快速干道前期工作。以产业为载体，加速济东工业集聚区建设，引导济宁高新区、兖州工业园区和邹城开发区错位发展，增强区域协作配套能力。

推进小城镇和美丽乡村建设。支持22个重点小城镇做大做强，集中培育7个小城镇示范点，全面深化城镇驻地环境综合整治。开展农村垃圾减量化试点和美丽庭院创建活动。抓好农村改水改厕，开展农村集中连片供水试点，完成20万户改厕任务。提升乡村绿化水平，新建30个市级绿化样板村、5个市级绿化样板镇，建设27个美丽乡村示范片区和5大示范带。

（四）加快建设生态济宁。以创建国家生态保护与建设示范区为统领，启动新一轮生态建设“三年行动计划”。强力推广清洁燃烧等治霾措施，持续开展大气污染防治“十大攻坚”行动，深化重点行业工业废气污染治理，加快推进燃煤机组及锅炉超低排放，完成5家电厂8台燃煤机组超低排放改造任务。加大化工、机械等行业挥发性有机物治理，加快鲁抗医药等企业退城进园步伐。严厉打击建筑工地扬尘污染和渣土运输抛洒。抓好黄标车清理、秸秆禁烧等工作。完善“治用保”水污染防控体系，加强沿河环湖污染源治理，加快人工湿地水质净化、中水回用和雨污分流管网建设，确保南四湖水质稳定达标。强化节能减排刚性指标管理，坚决杜绝新上高耗能、高排放项目。开展百户企业节能低碳行动，创建循环经济示范园区。提高全民节能意识，鼓励绿色出行，支持新能源汽车推广应用。加大生态安全治理，实施山水林田湖生态保护和修复工程，划定环南四湖大生态带红线，完成湿地保护修复10万亩。开展平原水库、生态湿地建设试点，加快国家级光伏发电示范基地建设，综合治理塌陷地2.2万亩。巩固绿化模范城市创建成果，新增造林15万亩、绿色通道300公里。

（五）加快文化强市首善之区建设。一是大力弘扬优秀传统文化。全力推进曲阜文化建设示范区、孔子学院总部体验基地、干部政德教育基地、儒学人才高地建设。高标准推进孔子博物馆、“三孔”彩绘等文化项目。实施乡村儒学、社区儒学、孔子学堂、尼山书院推进计划，打造“儒学原乡·文化圣地”品牌。二是做大做强文化产业。搞好文化项目策划包装，搭建融资、担保、创意“三大平台”，用好文化旅游产业发展基金，增强文化产业发展动力。创新文化产业体制机制，建设专业化文化交易市场，提速推进尼山圣境等文化产业项目。以突破文化创意产业为引领，加速文化与科技、金融深度融合，大力发展国学研修等新型文化业态，拓展与海外孔子学院联系，打造儒家文化国际旅游目的地。加强文化遗产保护，推进历史文化遗产的传承和复兴。加快邹鲁文化生态保护实验区规划建设。三是大力发展公共文化。力争完成市文化中心主体工程，市杂技城建成使用。改善县级博物馆、图书馆、文化馆办馆条件，促进公共文化服务设施向社会免费开放。加快基层公共文化服务中心建设，继续实施“千场大戏进农村”等文化惠民工程，丰富城乡居民文化生活。

（六）不断提升民生保障水平。持续加大民生投入，全力办好惠民实事，进一步增强人民群众幸福感、满意度。

突出抓好精准扶贫。以脱贫攻坚“六大行动”为抓手，通过增加就业岗位、扶持产业发展、开发乡村旅游、政府兜底救助等方式，完成10.5万人的脱贫任务。加强贫困村基础设施建设，完成131个贫困村道路户户通、42个贫困村电网升级改造任务，解决20个贫困村饮水安全问题。设立扶贫开发公益基金，建立扶贫小额信贷服务平台，动员社会力量广泛参与扶贫事业，助力贫困户尽快脱贫致富。

着力提高教育质量。实施基础教育高水平均衡发展“三年行动计划”，加快基础教育综合改革，全面推开中小学校长职级制和去行政化。强化师资队伍建设，加大“名师名校长”培训和交流力度。改善义务教育薄弱学校基本办学条件，新建、改扩建城镇中小学90所、面积100万平方米，新增学位4万个，努力解决中小学大班额问题。落实中小学教师编制“县管校聘”。加大贫困学生救助力度。支持驻济高校加快发展，大力提升职业教育水平，推进产教融合、校企合作。

加快建设健康济宁。实施医疗卫生健康惠民“三年行动计划”。推进医药卫生体制和公立医院改革，全面推行分级诊疗，加强城乡卫生基础设施和人才队伍建设，促进医疗资源向社区、向农村流动。鼓励社会力量兴办医疗卫生事业，加快市第一人民医院高新区分院二期工程建设，力争市中医院新院区投入使用。全面落实一对夫妇生育两个孩子政策，优化出生人口性别比。扎实推进食安济宁建设。深入开展全民健身运动，办好第三届老年人运动会，加大健康知识普及，提高人民健康水平。

积极扶持就业创业。加快建设济宁创业大学，依托惠普、甲骨文人才实训基地，鼓励发展众创、众包、众扶、众筹，形成创业创新孵化载体新模式，力争每个县市区建成1处创客空间，新增孵化器面积20万平方米。启动实施终身职业技能培训计划，完成就业创业培训5.5万人，培训电商10万人，扶持1.2万人创业。

完善提升社保体系。推进“困难群体助保工程”，加强社会养老服务体系建设，妥善解决困难群体养老和医疗保障问题。启动实施全民参保登记计划，调整企业退休人员养老保险待遇，提高城乡居民基础养老金、城乡低保、农村五保和职工基本医疗保险等标准。落实困难残疾人生活补贴和重度残疾人护理补贴，年内为100名听障儿童实施人工耳蜗植入手术。加强社会救助体系建设，倡导社会慈善捐助。

强化社会治理创新。推进社会信用体系建设，打造“信用济宁”品牌。深化“四社联动”，加强基层社会治理，建成100个农村社区服务中心市级示范点。深入开展社会矛盾大排查大调解，推行“阳光信访”，推动矛盾就地化解。加强舆情监管，提升舆论引导能力。完善立体化治安防控体系，推进平安济宁建设。健全自然灾害和突发事件应急机制。严格落实安全生产责任制，抓好重点领域安全整治，坚决遏制各类安全事故发生。做好第三次农业普查工作。加强国防动员、人民防空、民兵预备役建设和双拥工作。统筹做好民族宗教、外事侨务、对台和老龄工作，提升物价、统计和防震减灾等各项工作水平。

四、全面加强政府自身建设

新形势新任务新常态对政府工作提出更高要求。我们必须加快法治政府和服务型政府建设，全面提升依法履职能力，为实现“四大目标”提供有力保障。

严格依法行政。牢固树立法治思维、法治理念，善于运用法律手段解决实际问题，实现政府活动全面纳入法治轨道。自觉接受人大法律监督、政协民主监督，坚持重大事项向人大报告、政协通报制度，认真听取代表、委员及各民主党派、工商联、无党派人士和人民团体的意见建议。严格执行《济宁市重大行政决策程序规定》，健全公众参与、专家论证、风险评估、合法性审查制度，落实重大决策终身责任追究制度及责任倒查机制。完善综合行政执法体制，规范行政执法行为。强化社会监督、舆论监督和行政监督，推进审计监督全覆盖。

深化政务公开。围绕决策、执行、管理、服务、结果公开，加大政府信息公开力度，突出抓好公共资源配置、重大建设项目实施等政府信息公开。强化信息发布，做好社会热点回应。抓好互联网政务信息服务平台、便民服务平台建设，提升政府网站服务水平。

加强作风建设。弘扬实干和担当精神，完善工作责任制，深化“庸懒散”专项治理，解决不愿为、不会为、不敢为问题。开展绩效机关创建活动，优化公共服务流程，提升工作效能。进一步精简会议文件，积极推行一线工作法，力促问题在一线解决。坚持全年任务目标化、所有目标项目化、每个项目责任化、每项责任考核化，对重点工作、重要事项、重大工程一律实行项目制管理，成立专门项目部，实体运作，挂图作战，严格考核，确保各项任务落到实处。

建设清廉政府。严守政治纪律和政治规矩，巩固拓展党的群众路线教育实践活动成果，完善“三严三实”作风建设长效机制。认真落实党风廉政建设责任制，健全完善惩治和预防腐败体系，坚持用制度管权、管人、管事，把权力关进制度的笼子。厉行勤俭节约，继续压减“三公经费”等一般性支出，腾出更多资金用于基本公共服务。加大惩治腐败力度，严肃查处各种侵害群众利益的不正之风和腐败行为，营造干部清正、政府清廉、政治清明的良好环境。

各位代表，宏伟目标鼓舞人心，美好前景催人奋进，承载着850万济宁人民的梦想和期盼，我们踏上了“十三五”发展的新征程。让我们更加紧密地团结在以习近平同志为总书记的党中央周围，在中共济宁市委的坚强领导下，同心同德，开拓进取，为加快建设经济文化强市，夺取全面建成小康社会的伟大胜利而努力奋斗！
